# Supplementary material for: A Unique Relative of Rotifer Birnavirus Isolated from Australian Mosquitoes
Source: Viruses. 2020 Sep 22;12(9):1056. doi: 10.3390/v12091056 (PMC7552023; doi:10.3390/v12091056)
Supplement: Supplementary file 1 [file viruses-12-01056-s001.zip › Revised suplemenrary figures and files/Supplementary tables and figures proofed.docx]

| Primer name | Sequence (5’ – 3’) | Target | Reference |
| --- | --- | --- | --- |
| NidoF1 | GTTGTATGCTATGCCGYCG | Alphamesonivirus | PMID: 24670468 |
| NidoR1 | TCCATAGTATCGTAGCAATTCC |  |  |
| CORV-like_F | TTATCGGCAGACGGGATTCG | Parry’s Lagoon virus | PMID: 27213426 |
| CORV-like_R | CGCTTTCGTTAGCACCATCG |  |  |
| FU2 | GCTGATGACACCGCCGGCTGGGACAC | Flavivirus NS5 | PMID: 9420202 |
| cFD3 | AGCATGTCTTCCGTGGTCATCCA |  |  |
| CsV_Fwd | AGCCGTTATCAACTCTCTCG | Castlerea virus ORF1 | PMID: 28469377 |
| CsV_Rev | CGGTGAGAAGTCGATGAG |  |  |
| Sead_Seg10F | GTTATTTTTTCTAAGTGACA | Liao ning virus segment 10 | PMID: **29533743** |
| Sead_Seg10R | GCTAAGATTGTAAACACGGTT |  |  |
| Birna_VP1F2 | TGGTACTCTCTAGACTTAGTTCG | ABV RdRP (VP1, segment B) | This paper |
| Birna_VP1R | TTCTCCATAGCACGATTCACTGC |  |  |
| Birna_StrF | TAGTCTTCGTGACCAAGG | ABV polyprotein (segment A) | This paper |
| Birna_StrR | ATGTGGCTAGTCTTTCAGC |  |  |

**Table S1:** Primer sets used for RT-PCR screening


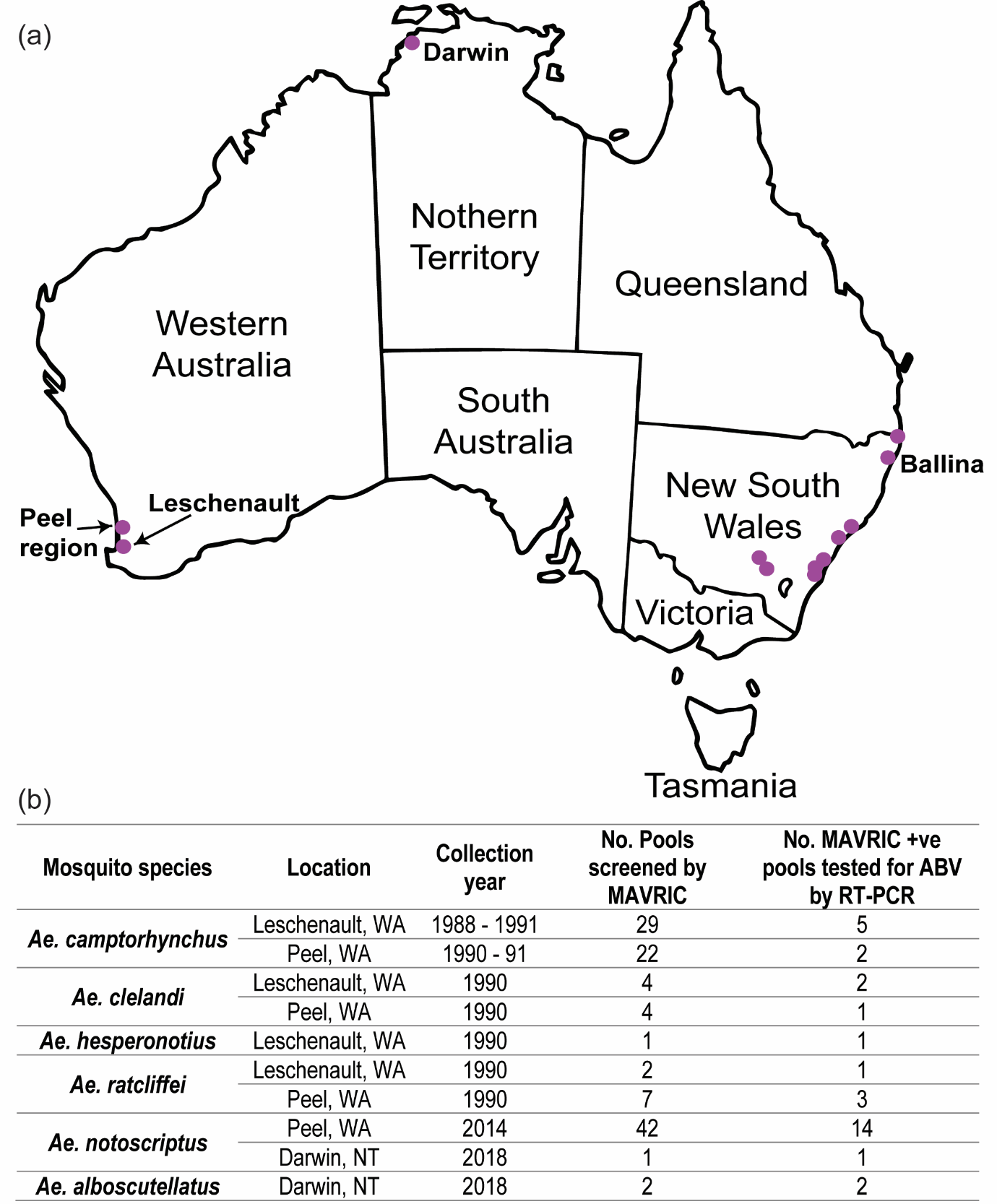


**Figure S1**. (a) Map of mosquito collection sites described in this paper. Magenta dots depict approximate regions where mosquitoes where collected. (b) Summary of MAVRIC-positive mosquito pools collected in the Northern Territory and Western Australia tested for ABV.


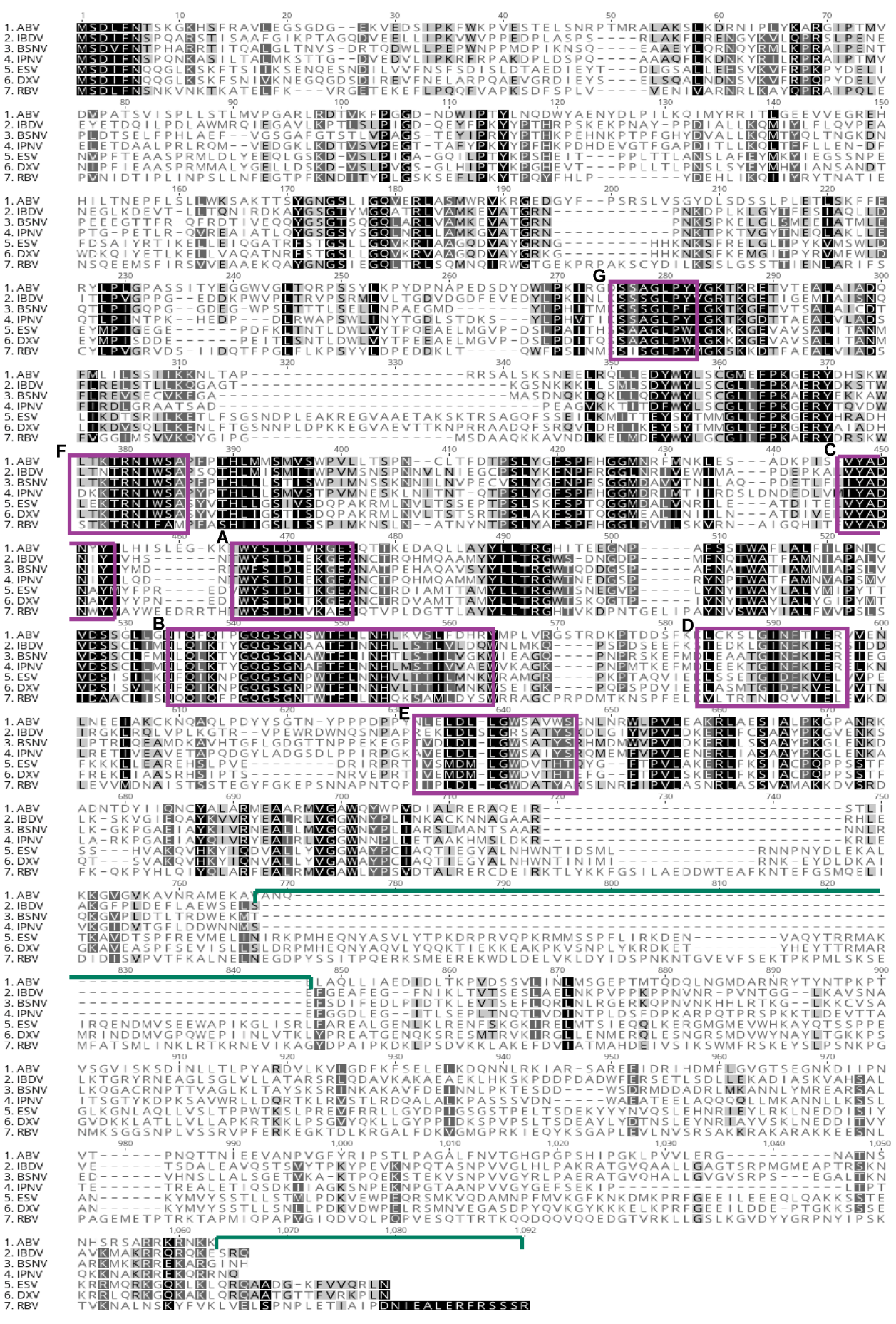


**Figure S2.** Clustal W alignment of amino acid VP1 sequences for ABV, RBV (CAX33877), IBDV (ANY27027), BSNV (CAD30689), ESV (AEW87521), DXV (NP_690836) and IPNV (NP_047196). Conserved motifs are indicated by magenta boxes. C-terminal domain insertions found in RBV and entomobirnaviruses are indicated by green brackets.


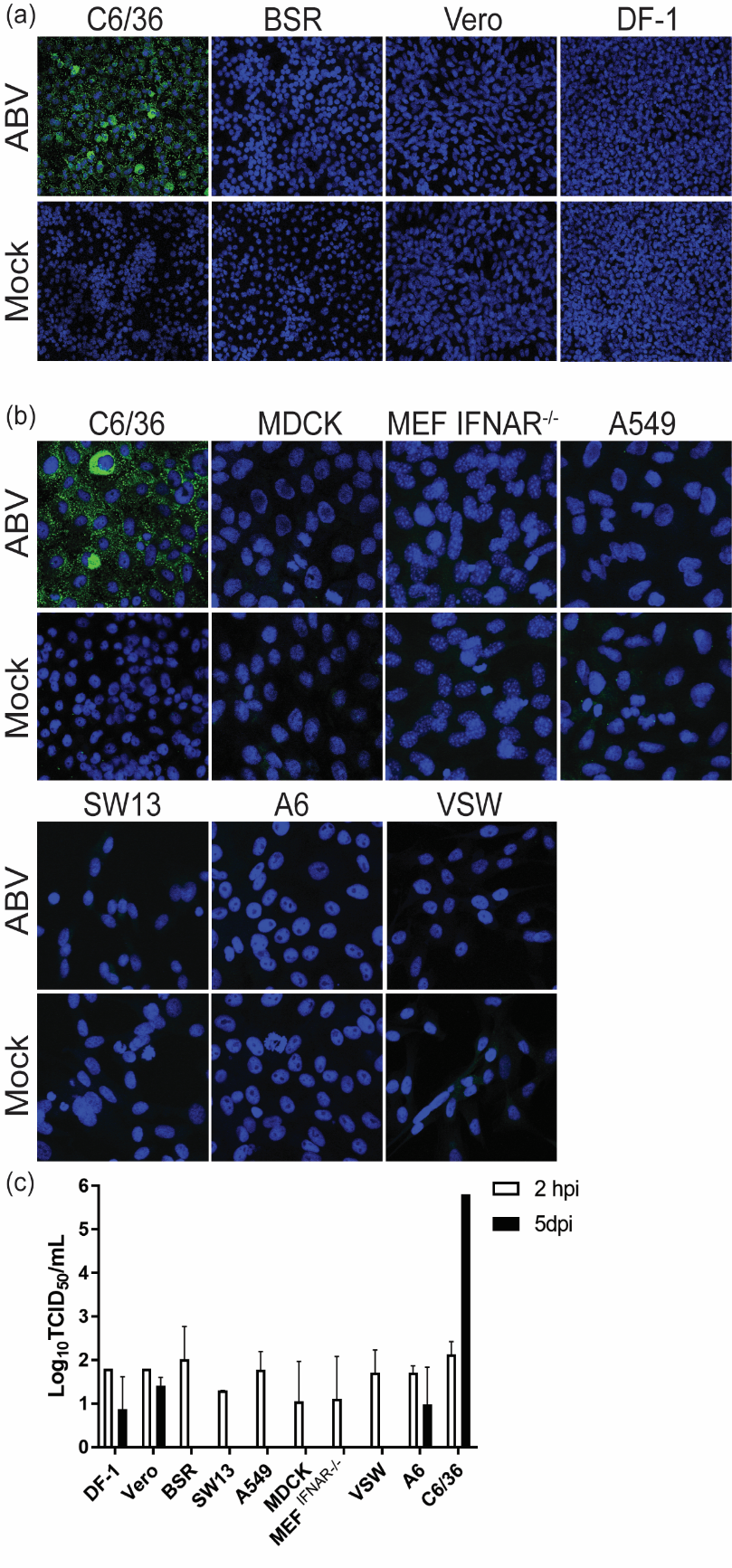


**Figure S3.** Cells fixed 5 days post-inoculation with ABV and stained for double-stranded RNA (green), nuclei (blue). (a) Baby hamster kidney (BSR), African green monkey (Vero), Chicken embryo fibroblast (DF-1) and *Ae. albopictus* (C6/36) cell images taken at 20x magnification. (b) Human (SW13, A549), Madin-Darby canine kidney (MDCK), interferon-α/β receptor-deficient, mouse embryonic fibroblast; VSW, Russel’s viper spleen tumour; A6, South African clawed toad kidney epithelial and *Ae. albopictus* (C6/36) cell images taken at 40x magnification. (c) Mean titres of ABV derived from supernatants harvested from vertebrate or C6/36 cells (positive control) at 2 hours (white) and 5 days (black) post-infection. Error bars represent standard deviations between three replicates.
