# Supplementary figures and images for: A Unique Relative of Rotifer Birnavirus Isolated from Australian Mosquitoes
Source: Viruses. 2020 Sep 22;12(9):1056. doi: 10.3390/v12091056 (PMC7552023; doi:10.3390/v12091056)

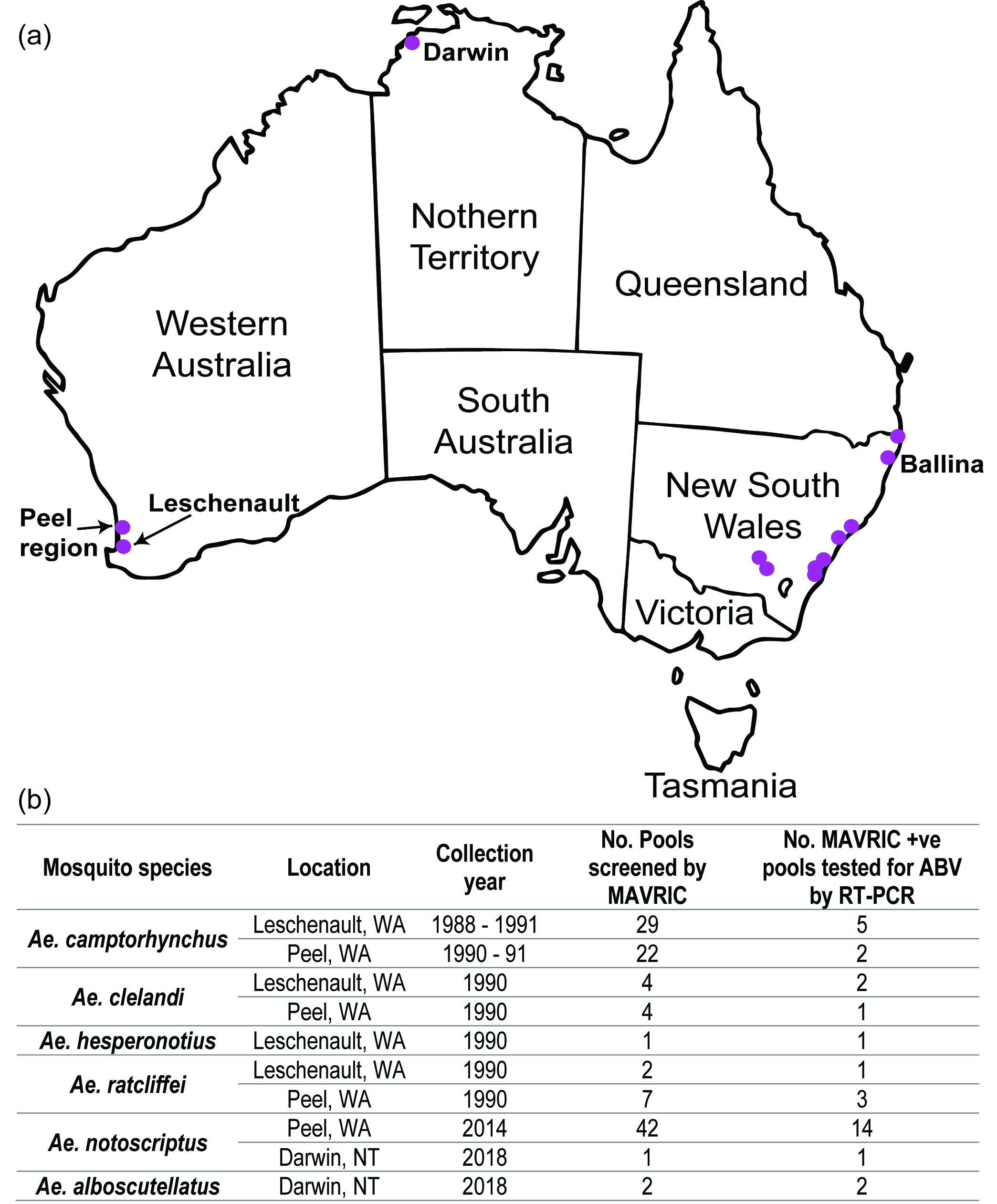

Supplement: Supplementary file 1 [file viruses-12-01056-s001.zip › Revised suplemenrary figures and files/Figure S1 - collection sites and additional screening.tif]

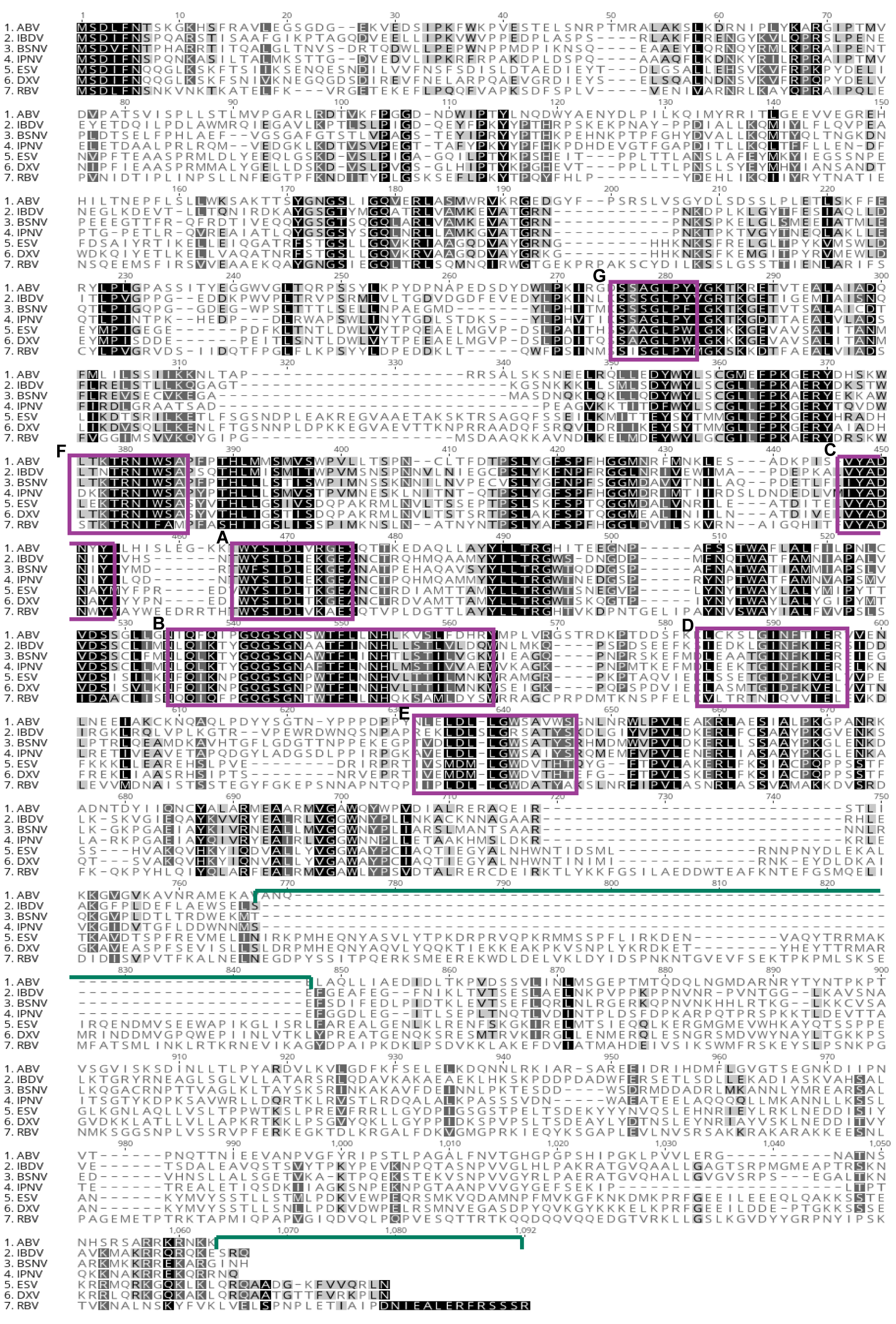

Supplement: Supplementary file 1 [file viruses-12-01056-s001.zip › Revised suplemenrary figures and files/Figure S2 - rdrp alignment.tif]

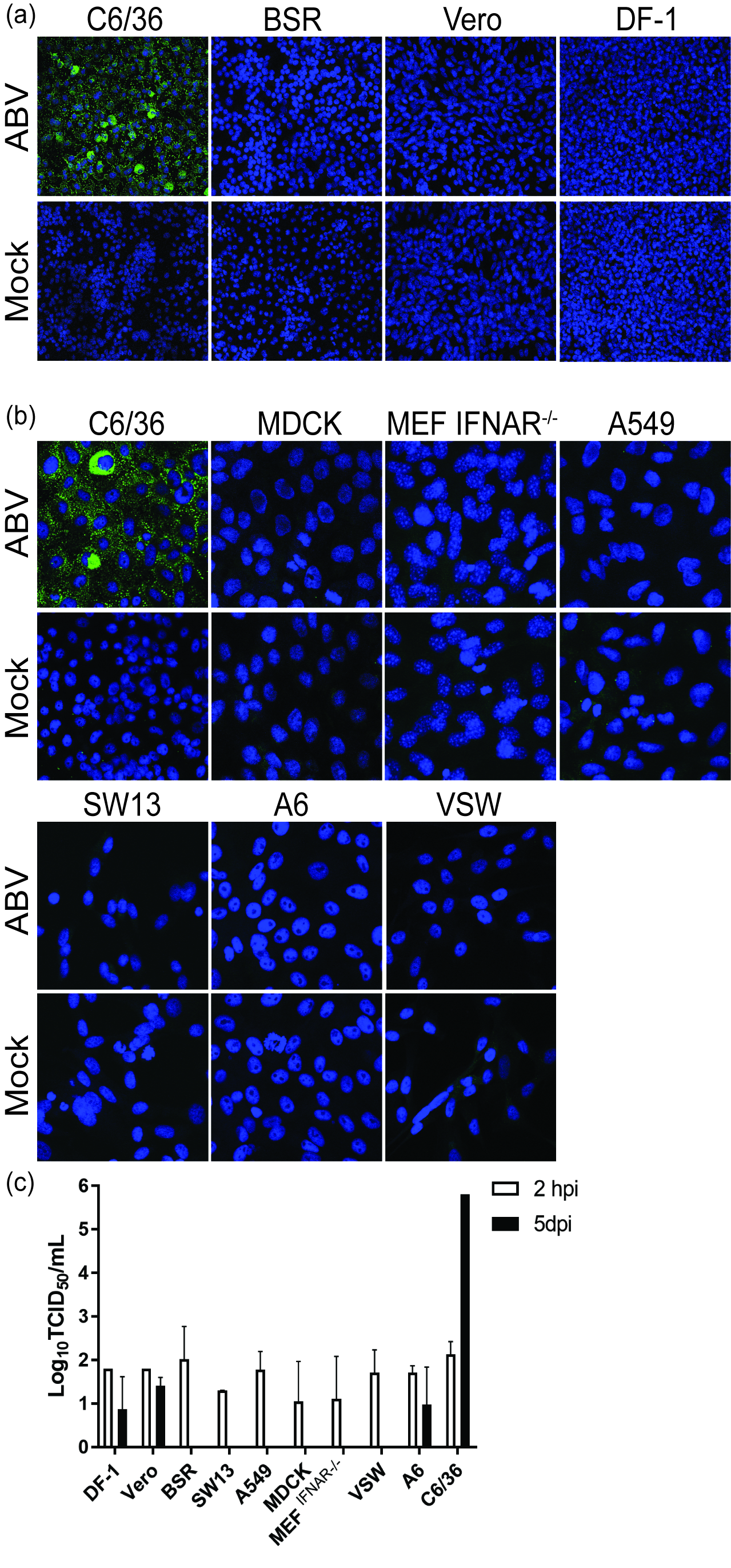

Supplement: Supplementary file 1 [file viruses-12-01056-s001.zip › Revised suplemenrary figures and files/Figure S3 - vertebrate cell assay.tif]
